# Monoclonal plasma cells with normal phenotype can lead to diagnosis of small B‐cell lymphoma

**DOI:** 10.1002/jha2.540

**Published:** 2022-09-30

**Authors:** Alban Canali, Jill Corre, Brigitte Riviere, Pauline Condom, Jean‐Baptiste Rieu

**Affiliations:** ^1^ Haematology Laboratory Cancer University Institute of Toulouse ‐ Oncopole Toulouse France; ^2^ Medical Biology Laboratory, Centre Hospitalier Intercommunal Castres ‐ Mazamet Castres France

1

A 66‐year‐old man was referred for non‐regenerative anaemia (Hb 114 g/L) and monoclonal IgG Kappa gammopathy (29.9 g/L). Biochemical test results showed normal serum creatinine and calcium and slight increase beta2‐microglobulin (3.21 mg/L). Bone marrow examination revealed 3% plasma cells (Figure 1A May‐Grünwald‐Giemsa, ×100 objective, blue arrows) and 9% lymphocytes (Figure 1A red arrows) without morphological changes related to malignancy. Flow cytometric immunophenotyping showed CD38‐positive, CD138‐positive plasma cells with Kappa monotypic expression and without aberrant expression of CD45, CD19, CD117, CD56, CD81 and CD27 (Figure 1B). Next generation sequencing of sorted plasma cells revealed *MYD88* L265P mutation (VAF 0.22). These results were not in favour of monoclonal gammopathy of undetermined significance (MGUS) and led us to look for B‐cell lymphomas. Flow cytometric immunophenotyping of lymphocytes, showing Kappa monotypic B‐cells population (Figure 1B) with coexpression of CD19, CD20, CD79a and without CD5, CD23, CD10, was finally indicative of lymphoplasmacytic lymphoma.

**FIGURE 1:**
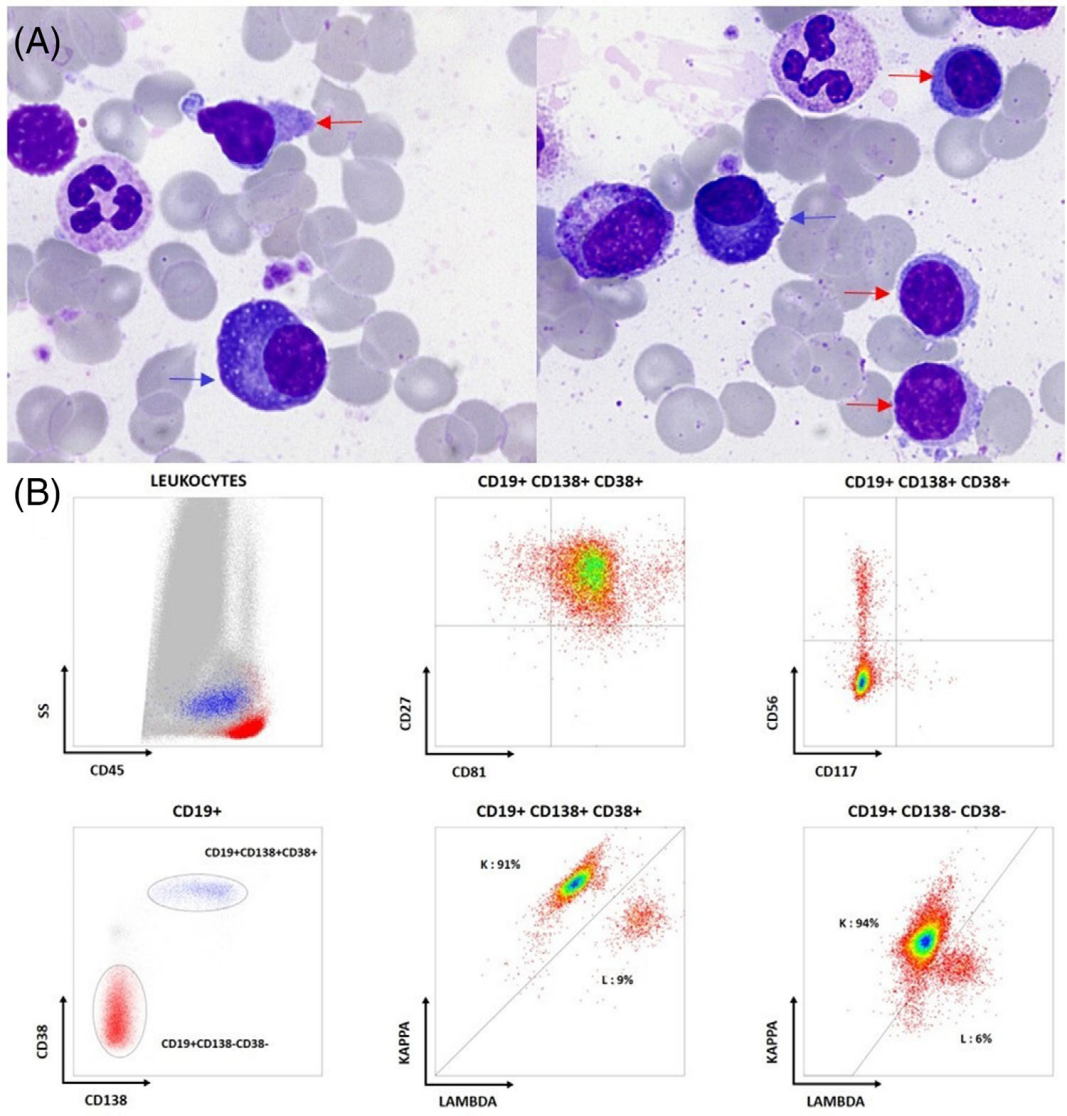
(A) Bone marrow examination, May‐Grünwald‐Giemsa, ×100 objective. Plasma cells (blue arrows) and lymphocytes (red arrow) of normal morphology. (B) Flow cytometric immunophenotyping of plasma cells and B‐cells in bone marrow sample. Plasma cells (CD19+ CD138+ CD38+) with Kappa monotypic expression and phenotype profile similar to that of normal plasma cells (CD45+, CD19+, CD27+, CD81+, CD56−, CD117−). B‐cells (CD19+ CD138− CD38−) with Kappa monotypic expression

Plasmacytic differentiation in B‐cells lymphomas is a well‐known phenomenon [1, 2] that can be challenging for differential diagnosis, especially with MGUS and multiple myeloma (MM). In MGUS and MM, plasma cells typically exhibit aberrant phenotype with loss of CD19 and CD45, expression of CD56 and/or CD117 and/or lack of CD81 and/or CD27 [3, 4]. Thus, the presence of a monoclonal population of plasma cells with a normal phenotype should prompt us to look for B‐cells lymphomas [5].

## AUTHOR CONTRIBUTIONS

AC, JC and JBR wrote the paper. JBR took the pictures. BR and PC performed the bone marrow examination. AC and JBR performed flow cytometric studies, and JC performed molecular studies.

## CONFLICT OF INTEREST

The authors have no competing interests.

## ETHICS STATEMENT

This manuscript respects ethic policy of CHU Toulouse for treatment of human research participants. The authors did not obtain written informed consent from the patient but the patient did not object to his data being used for research purposes (as required by ethic policy of CHU Toulouse).

## Data Availability

Data sharing is not applicable to this article as no new data were created or analyzed in this study.
